# Comparison of the inflammatory biomarkers IL- 6, TNF-α, and CRP to predict the effect of nutritional therapy on mortality in medical patients at risk of malnutrition: A secondary analysis of the randomized clinical trial EFFORT

**DOI:** 10.1186/s12950-025-00442-0

**Published:** 2025-04-24

**Authors:** Carla Wunderle, Elisabeth Martin, Alma Wittig, Pascal Tribolet, Thomas A. Lutz, Christina Köster-Hegmann, Zeno Stanga, Beat Mueller, Philipp Schuetz

**Affiliations:** 1 Medical University Department, Division of General Internal and Emergency Medicine, Division of Endocrinology, Diabetes and Metabolism, Kantonsspital Aarau, Tellstrasse 25, Aarau, 5001 Switzerland; 2 Medical Faculty, University of Basel, Basel, 4056 Switzerland; 3 Department of Health Professions, Bern University of Applied Sciences, Bern, 3008 Switzerland; 4 Department of Nutritional Sciences, Faculty of Life Sciences, University of Vienna, Josef-Holaubek-Platz 2, Vienna, 1090 Austria; 5 Institute of Veterinary Physiology, University of Zurich - Vetsuisse Faculty, Zurich, 8057 Switzerland; 6 Division of Diabetes, Endocrinology, Nutritional Medicine, and Metabolism, Bern University Hospital, University of Bern, Bern, 3010 Switzerland

**Keywords:** IL- 6, TNF alpha, CRP, Inflammation, Mortality, Nutritional therapy, Clinical outcomes, Polymorbid, Individualization

## Abstract

**Background:**

Inflammation is a key driver of disease-related malnutrition and patients with high inflammation may not show the same benefits from nutritional therapy as other patients. We compared in an exploratory manner the prognostic ability of interleukin- 6 (IL- 6), tumor necrosis factor-alpha (TNF-α) and C-reactive protein (CRP) to predict outcome and response to nutritional therapy, respectively, within a large cohort of patients from a previous nutritional trial.

**Methods:**

This is a secondary analysis of the Swiss-wide, multicenter, randomized controlled *Effect of early nutritional therapy on Frailty*,* Functional Outcomes*,* and Recovery of malnourished medical inpatients Trial* (EFFORT) trial comparing individualized nutritional support with usual care nutrition in medical inpatients. The primary endpoint was 30-day all-cause mortality.

**Results:**

We included 996 patients with an overall mortality rate of 6% within 30 days. Compared to patients with low IL- 6 level < 11.2pg/mL, patients with high levels had a more than 3-fold increase in mortality at 30-days (adjusted HR 3.5, 95% CI 1.95–6.28, *p* < 0.001), but tended to have a less pronounced mortality benefit from individualized nutritional therapy as compared to usual nutritional care (hazard ratio 0.82 vs. 0.32). CRP and TNF-α were not associated with mortality, but patients with increased CRP levels > 100 mg/dl also showed a trend towards a diminished response to nutritional intervention (hazard ratio 1.25 vs. 0.47).

**Conclusion:**

Our findings support the thesis that a high inflammatory state is linked to reduced benefits from nutritional therapy. Apparently, CRP and IL- 6 effectively predict treatment response, but IL- 6 may additionally serve as a prognostic marker for increased mortality. This finding might help to develop improved treatment strategies for patients with elevated inflammatory profiles.

**Trial registration:**

Clinicaltrials.gov as NCT02517476 (registered 7 August 2015).

**Supplementary Information:**

The online version contains supplementary material available at 10.1186/s12950-025-00442-0.

## Introduction

Malnutrition affects 20–50% of hospitalized patients, and increases morbidity, mortality, and physical decline among this patient population [1–3]. Inflammation is a key driver of malnutrition acting through multiple pathways [4, 5]. During inflammation, the body mobilizes internal energy reserves via lipolysis, glycolysis, glycogenolysis, gluconeogenesis, and proteolysis to supply energy for immune activation, tissue repair, and vital organ function. This increases internal energy production, combined with ongoing catabolism and peripheral insulin resistance [6, 7]. Inflammation also affects appetite and gut function, ultimately leading to weight loss, sarcopenia, and malnutrition over time [2, 8].

Several trials and meta-analyses found individualized nutritional therapy to be beneficial for patients with malnutrition in the hospital setting [9–13]. The Effect of Early Nutritional Support on Frailty, Functional Outcomes, and Recovery of Malnourished Medical Inpatients (EFFORT) trial found that among medical multimorbid inpatients, individualized nutritional therapy reduces the risks for complications and mortality [9]. However, there is growing concern that patients with high inflammation may not have the same benefits from nutritional therapy as other patients. In fact, several large-scale studies from the intensive care unit (ICU) [14, 15] and subgroup analyses of more acutely but not severely ill medical patients [16, 17] showed no benefit from full trophic nutritional feeding [18–20]. Also, a secondary analysis of the aforementioned EFFORT trial suggested that patients with high inflammation, as indicated by C-reactive protein (CRP) levels above 100 mg/dL, did not experience the same mortality benefit from nutritional intervention compared to the overall patient cohort [16].

Today, the prognostic value of CRP has been studied in polymorbid patients at nutritional risk, but as a single biomarker of inflammation, it has certain limitations, such as high inter-individual variability, inconsistent hepatic CRP release in response to stimuli, and fluctuations from day to day [21–23]. Consequently, assessing additional inflammatory markers such as interleukin 6 (IL- 6) and tumor necrosis factor alpha (TNF-α), along with consideration of the timing of their peak plasma concentrations, may provide a more comprehensive evaluation of inflammation and improve clinical insights for predicting treatment response in nutritionally at-risk patients [21, 22]. The pro-inflammatory cytokines IL- 6 and TNF-α are directly released in response to various stressors [6], such as illness, and they stimulate the liver to produce acute-phase proteins like CRP. Due to this inflammatory cascade, cytokine plasma concentration peaks occur at different times than those of acute-phase proteins. IL- 6 and TNF-α reach their highest plasma levels within 90 to 120 min, whereas CRP peaks 1–2 days after the initial trigger [22, 23]. Herein, we performed a secondary analysis of EFFORT [9] to compare the prognostic ability of the inflammatory markers IL- 6, TNF-α and CRP to predict, firstly, clinical outcome and secondly, the response to nutritional therapy.

## Materials and methods

### Study design and setting

This is a secondary analysis of EFFORT, a pragmatic, open-label, investigator-initiated randomized control trial (RCT) carried out in eight Swiss hospitals [9]. The original EFFORT study was conducted between April 2014 and February 2018, investigating the impact of early, individualized nutritional support on clinical outcomes of medical inpatients at nutritional risk. Following the initial EFFORT study, cytokine levels of IL- 6 and TNF-α were measured in biobank samples of patients in collaboration with the Institute of Veterinary Physiology at the University of Zurich (UZH) from June 2023 to July 2024. The Ethical Committee of Northwestern Switzerland approved the study protocol in January 2014 (EKNZ; 2014_001). The protocol, the main results of the EFFORT trial along with various secondary analyses have been published elsewhere [9, 24, 25].

### Patient population

To be included in the initial EFFORT study, patients had to have a nutritional risk total score of at least 3 points, according to the Nutritional Risk Screening 2002 (NRS) [26], have an expected length of stay (LOS) of at least 5 days and were willing to give their informed consent. Patients were excluded if they were admitted to the intensive care unit (ICU) or surgical unit, received nutritional support at admission, were unable to ingest oral nutrition, or had previously participated in the trial. Additionally, exclusion criteria included the presence of certain diseases (anorexia nervosa, acute pancreatitis, acute liver failure, or cystic fibrosis), being terminally ill, undergoing gastric bypass surgery or stem cell transplantation, or having contraindications for nutritional support [9]. For this secondary analysis, our patient population represented six Swiss hospitals (University Clinic of Aarau, University Hospital of Bern, Cantonal Hospitals of Muensterlingen, St. Gallen and Lucerne and Regional Hospital of Solothurn) for which IL- 6, TNF-α and CRP measurements were available.

### Nutritional intervention

Randomization was done with an interactive web-response system, with variable block sizes, and patients were stratified according to site and the severity of malnutrition. The intervention group received nutritional therapy, which was initiated within 48 h of hospital admission. An individual plan for each patient was developed by trained registered dietitians based on present international guidelines [27]. For all intervention group patients, regardless of their baseline inflammation, that did not achieve at least 75% of their daily energy and protein targets within 5 days, therapy was escalated to additional enteral nutrition and, if necessary, to additional parenteral nutrition. Therapy was extended until 75% of nutritional target were achieved, with the composition of the oral, enteral and parenteral nutrition forms being individually tailored. Patients in the control group received usual hospital food without daily energy and protein targets and with no nutritional counselling. Further details of the procedures were published in the main article [9].

### Analysis of blood biomarkers

Blood samples were collected upon study inclusion, immediately processed and frozen in aliquots at the controlled temperature of − 80 °C for later analysis. IL- 6 and TNF-α were analyzed from June 2023 to July 2024, amounting to 997 samples at the Institute of Veterinary Physiology at the University of Zurich. The plasma cytokine levels were measured in 1:1 diluted samples using a self-assembled MSD Multi-Spot Assay System MESO Scale *U-PLEX Human IL- 6 Assay* and *U-PLEX Human TNF-α Assay*, respectively (MSD Maryland, USA). The concentrations of CRP were taken from the hospital’s routine laboratory analysis and were not remeasured. As a result, measured values are not available for the entire cohort. The laboratory personnel were blinded to the randomization allocation.

### Endpoints

The primary endpoint for this analysis was all-cause mortality measured at 30 days. Our secondary endpoints included all-cause mortality over medium to long-term periods (at 180 days, 1 year, 2 years, 3 years, 5 years). Additional secondary endpoints were major complications, adverse events, quality of life, and admission to the ICU within 30 days, a decline in functional status of more than 10%, as measured by Barthel Index [28] at hospital admission and after 180 days, and non-elective hospital readmission after discharge. We also examined length of hospital stay.

### Statistical analysis

STATA 17.0 was used for statistical analysis. Statistical significance was tested at 95% confidence intervals (CI), corresponding to a *p*-value of 0.05. We performed descriptive statistics for baseline characteristics with continuous variables being expressed as mean ± standard deviation (SD), and binary and categorical variables were expressed as the number or count and percentages. We compared baseline characteristics between high and low levels of cytokines. The 2-sample t-test was used for continuous, normally distributed, and the Pearson’s chi-squared test for categorical and binary variables. We used qnorm plots to visually check for normality. Univariate and multivariate linear regressions were performed to analyze associations with baseline characteristics, malnutrition parameters, primary diagnoses, and comorbidities. The malnutrition parameters included anamnestic information on food intake, weight development and the treating physician’s assessment of the severity of the disease using a score between 0 and 3. These parameters were determined upon admission to the hospital using the NRS and, in the case of food intake, referred to the week prior to hospitalization, and in the case of weight development, to the three months prior to hospitalization. Adjustments were applied for possible confounding factors. We excluded outliers that surpassed a value of mean ± 3 standard deviations of the sample (z-score method) for linear regression models, to mitigate skewness and enhance the robustness of the analysis [21]. Specifically, we excluded 7 IL- 6, 4 TNF-α, and 19 CRP values for these analyses. Of the 997 values received, we excluded one outlier due to its value exceeding the measurable dynamic range of the assay kit. Our patient population was stratified into high and low levels for IL- 6 and TNF-α, respectively. To determine the empirical optimal cutoff values for our stratification, we performed a statistical cut-point analysis using the Liu method via Receiver Operating Characteristic (ROC) for the primary endpoint (30-day mortality). This amounted to a cutoff concentration of 11.17pg/mL for IL- 6 at an AUC of 0.673 and the respective cutoff concentration for TNF-α of 2.78pg/mL at AUC 0.546. We stratified patients according to CRP concentration consistent with previous investigations [16]. In particular, patients with a CRP concentration of over 100 mg/dl on admission were counted as belonging to the high CRP group.

Associations between high versus low cytokine concentrations and the primary clinical endpoint were evaluated using Cox regression models, secondary clinical endpoints were evaluated using both Cox and logistic regression models (for time-to-event and binary variables, respectively). Results of time-to-event variables were reported as hazard ratios (HR) and presented as Kaplan-Meier curves. Logistic regressions of secondary endpoints were reported as Odds ratios (OR). Adjustments were made for the following potential confounders: sex, age, baseline NRS, the most frequent diagnoses (cancer, infectious and cardiovascular disease, and frailty), and the most frequent comorbidities (chronic kidney disease and hypertension). Finally, we investigated the effect of nutritional therapy on 30-day mortality, stratifying by low compared to high baseline inflammatory marker. In order to understand whether the effect of nutritional support differs according to high vs. low inflammatory markers, we included interaction terms in the regression models and reported *p*-values.

## Results

### Patient characteristics

From the initial EFFORT trial population of 2028 patients, we had IL- 6 and TNF-α levels of 996 patients. A total of 492 were assigned to the intervention group (Supplemental Fig. 1) and 444 had high levels of IL- 6 (> 11.17 pg/mL) at admission. The mean age of the population was 72.7 years, and 535 (53.7%) patients were male. Overall, the most common main diagnoses were infectious diseases (31.1%), cancer diseases (14.3%), cardiovascular diseases (10.9%) and frailty (10.6%). There were some imbalances between the low and high IL- 6 groups at admission: Patients in the high IL- 6 group had a higher prevalence of infectious diseases (41.4%) compared to those in the low IL- 6 group (22.8%), whereas frailty was less prevalent in patients with high IL- 6 levels (7.4% compared to 13.2%) (Table 1). When stratified by CRP, the groups showed imbalances in NRS total score distribution and some main diagnoses. The baseline characteristics stratified according to TNF-α levels and CRP levels can be found in the appendix (Supplemental Tables 1 and 2).

**Table 1:** Baseline characteristics, stratified by low and high IL- 6 levels at admission

	**Overall**	**Low IL- 6**	**High IL- 6**	***p*** **-value**
**Sociodemographic info**				
Patient number, n (%)	996(100)	552 (55.4)	444 (44.6)	
Male sex, n (%)	535 (53.7)	275 (49.8)	260 (58.6)	0.006
Age, mean (SD)	72.7 (14.4)	72.7 (15.0)	72.8 (13.7)	0.88
**Nutritional assessment, mean (SD)**				
Body mass index (kg/m²)	24.7 (5.2)	24.6 (5.4)	24.8 (5.0)	0.65
Mean body weight (kg)	70.8 (17.0)	69.6 (16.9)	72.3 (16.9)	0.022
**NRS total score, n (%)**				0.066
3 points	334 (33.5)	193 (35.0)	141 (31.8)	
4 points	383 (38.5)	220 (39.9)	163 (36.7)	
5 points	238 (23.9)	123 (22.3)	115 (25.9)	
6 points	41 (4.1)	16 (2.9)	25 (5.6)	
**Intervention group**	492 (49.4)	278 (50.4)	214 (48.2)	0.50
**Admission diagnosis, n (%)**				
Infection	310 (31.1)	126 (22.8)	184 (41.4)	< 0.001
Cancer	142 (14.3)	77 (13.9)	65 (14.6)	0.76
Cardiovascular disease	109 (10.9)	67 (12.1)	42 (9.5)	0.18
Frailty	106 (10.6)	73 (13.2)	33 (7.4)	0.003
Lung disease	60 (6.0)	40 (7.2)	20 (4.5)	0.071
Gastrointestinal disease	91 (9.1)	51 (9.2)	40 (9.0)	0.9
Neurological disease	61 (6.1)	42 (7.6)	19 (4.3)	0.029
Renal disease	20 (2.0)	11 (2.0)	9 (2.0)	0.97
Metabolic disease	30 (3.0)	24 (4.3)	6 (1.4)	0.006
Other	24 (2.4)	14 (2.5)	10 (2.3)	0.77
**Comorbidity, n (%)**				
Hypertension	508 (51.0)	286 (51.8)	222 (50.0)	0.57
Malignant disease	283 (28.4)	135 (24.5)	148 (33.3)	0.002
Chronic kidney disease	285 (28.6)	150 (27.2)	135 (30.4)	0.26
Coronary heart disease	298 (29.9)	156 (28.3)	142 (32.0)	0.2
Diabetes	207 (20.8)	114 (20.7)	93 (20.9)	0.91
Congestive heart failure	168 (16.9)	102 (18.5)	66 (14.9)	0.13
COPD	160 (16.1)	91 (16.5)	69 (15.5)	0.69
Peripheral arterial disease	77 (7.7)	45 (8.2)	32 (7.2)	0.58
Stroke	74 (7.4)	37 (6.7)	37 (8.3)	0.33
Dementia	35 (3.5)	17 (3.1)	18 (4.1)	0.41

We compared frequencies using Person’s chi-squared test and continuous, normally distributed variables using a two-sample t-test. *NRS* Nutritional Risk Screening 2002, *COPD* Chronic obstructive pulmonary disease

### Association of patient characteristics and nutritional parameters with IL- 6, TNF-α and CRP

We investigated the association between inflammatory markers on admission and different nutritional parameters, which we took from the information collected from the NRS, and the medical history according to the diagnosis report. While CRP was associated with various nutritional parameters such as reduced dietary intake and weight loss, neither IL- 6 nor TNF-α showed a significant association with malnutrition risk parameters according to the NRS total score or its components. However, we did find a significant association between higher disease severity and elevated IL- 6 levels. There were also some differences in regard to underlying diagnoses and comorbidities as shown in Supplemental Table 3. However, these did not follow any clear pattern.

### Association of IL- 6, TNF-α and CRP on mortality and secondary outcomes

**Fig. 1:**
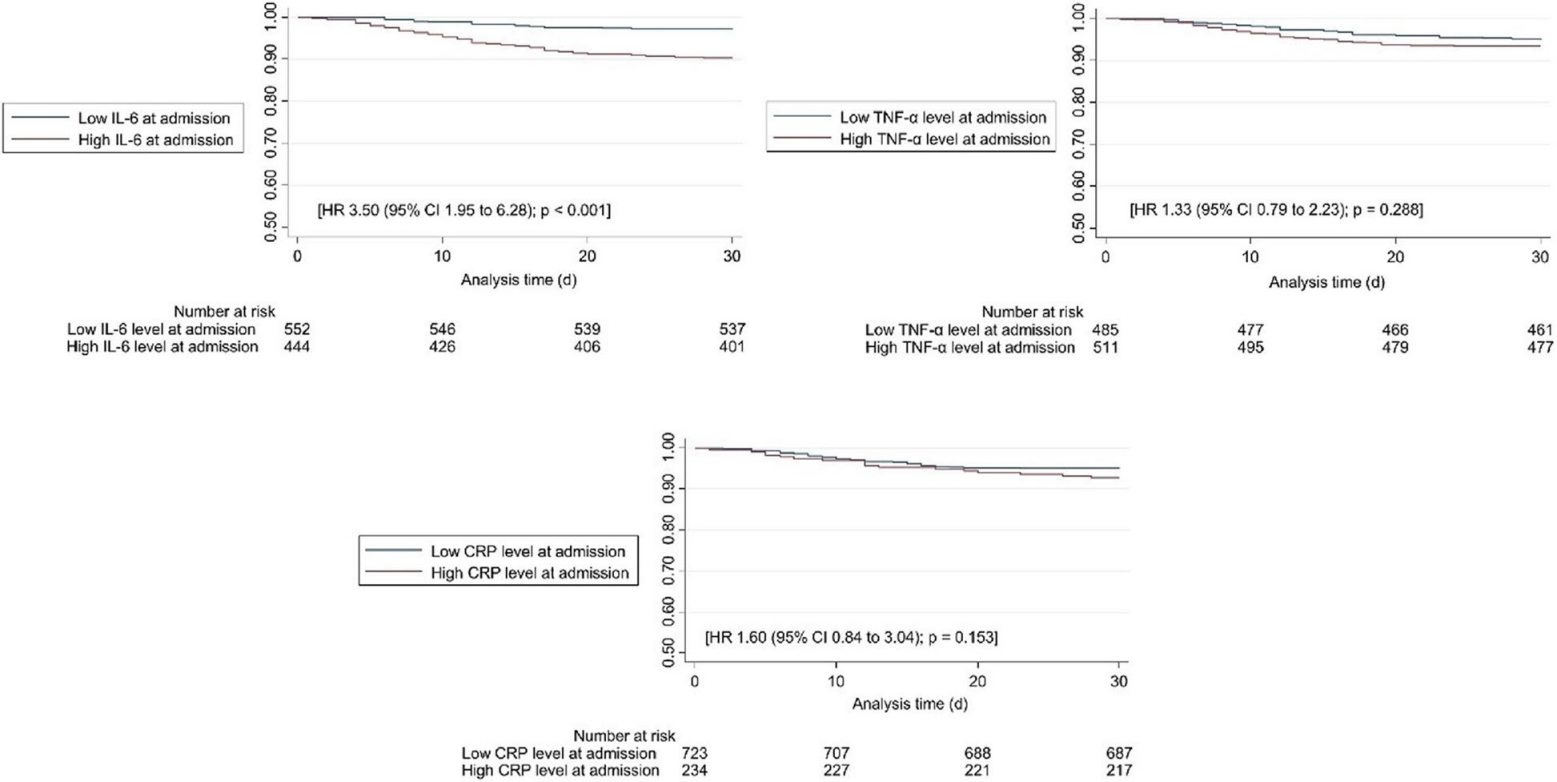
Kaplan-Meier survival estimates within 30-days comparing patients with high vs. low IL- 6, TNF-α and CRP levels. HR* was adjusted for age, sex, randomization group, Nutritional Risk Screening 2002 total score, the four most frequent main diagnoses (cancer, infection, cardiovascular disease, frailty), and the two most frequent comorbidities (hypertension, renal insufficiency). HR, hazard ratio; IL- 6, interleukin 6; TNF-α, tumor necrosis factor alpha; CRP, C-reactive protein

We further investigated the prognostic value of IL- 6, TNF-α and CRP regarding mortality at 30 days and other clinical outcomes. Overall, low IL- 6 levels had the strongest prognostic association in multivariate models adjusted for age, sex, randomization group, NRS, main diagnoses and comorbidities. Patients with high IL- 6 levels on admission had a more than 3-fold increased 30-day mortality risk with an adjusted HR of 3.5 (95% CI 1.95 to 6.28, *p*-value < 0.001) (Table 2). Similar results for IL- 6 were also found for mortality at longer follow-up times up to 5 years, with death rates remaining similar over the observation period, suggesting that this is more likely to be a short-term effect that persists. Prognostic results for TNF-α and CRP did not show significant associations at any time point. Kaplan-Meier survival graphs for 30-day mortality stratified by high compared to low biomarker levels are shown in Fig. 1.

**Table 2:** Prognostic value of high vs. low levels of IL- 6, TNF-α and CRP for mortality

**All-cause mortality**	**Events**		**Adjusted**	
	**Low plasma levels**	**High plasma levels**	HR* (95% CI)	***p*** **-value**
**30-day all-cause mortality**				
IL- 6	16/552 (2.9%)	43/444 (9.7%)	3.50 (1.95 to 6.28)	< 0.001
TNF-α	25/485 (5.2%)	34/511 (6.7%)	1.33 (0.79 to 2.23)	0.288
CRP	37/723 (5.1%)	17/234 (7.3%)	1.60 (0.84 to 3.04)	0.153
**180-day all-cause mortality**				
IL- 6	83/552 (15.0%)	115/444 (25.9%)	1.97 (1.47 to 2.63)	< 0.001
TNF-α	93/485 (19.2%)	105/511 (20.5%)	1.08 (0.82 to 1.44)	0.58
CRP	140/723 (19.4%)	45/234 (19.2%)	1.22 (0.84 to 1.77)	0.298
**1-year all-cause mortality**				
IL- 6	131/500 (26.2%)	157/411 (38.2%)	1.75 (1.38 to 2.23)	< 0.001
TNF-α	134/436 (30.7%)	154/475 (32.4%)	1.10 (0.87 to 1.39)	0.428
CRP	207/665 (31.1%)	64/211 (30.3%)	1.25 (0.91 to 1.71)	0.166
**2-year all-cause mortality**				
IL- 6	186/500 (37.2%)	207/411 (50.4%)	1.68 (1.37 to 2.06)	< 0.001
TNF-α	187/436 (42.9%)	206/475 (43.4%)	1.06 (0.87 to 1.29)	0.578
CRP	288/665 (43.3%)	86/211 (40.8%)	1.09 (0.83 to 1.43)	0.529
**3-year all-cause mortality**				
IL- 6	206/552 (37.3%)	221/444 (49.8%)	1.66 (1.37 to 2.02)	< 0.001
TNF-α	206/485 (42.5%)	221/511 (43.2%)	1.05 (0.87 to 1.27)	0.612
CRP	313/723 (43.3%)	93/234 (39.7%)	1.08 (0.84 to 1.41)	0.542
**5-year all-cause mortality**				
IL- 6	217/500 (43.4%)	227/411 (55.2%)	1.60 (1.32 to 1.94)	< 0.001
TNF-α	213/436 (48.9%)	231/475 (48.6%)	1.03 (0.86 to 1.25)	0.73
CRP	327/665 (49.2%)	95/211 (45.0%)	1.07 (0.82 to 1.38)	0.624

Groups are based on high or low cytokine levels. Data are number of events (%). HR* was adjusted for age, sex, randomization group, Nutritional Risk Screening 2002 total score, the four most frequent main diagnoses (cancer, infection, cardiovascular disease, frailty), and the two most frequent comorbidities (hypertension, renal insufficiency). *CI* Confidence interval, *HR* Hazard ratio, *IL- 6* Interleukin 6, *TNF-α* Tumor necrosis factor alpha, *CRP* C-reactive protein

Furthermore, high IL- 6 was associated with specific secondary clinical outcomes, but not with all those examined. Particularly, with higher risk of complications, longer length of hospital stays and greater decline in functional independence according to the Barthel Index. There were some individual associations with CRP and secondary results, but these appear to be coincidental. The results are presented in Supplemental Table 4.

### Predictive value of IL- 6, TNFα and CRP for the effect of nutritional therapy on mortality

**Fig. 2:**
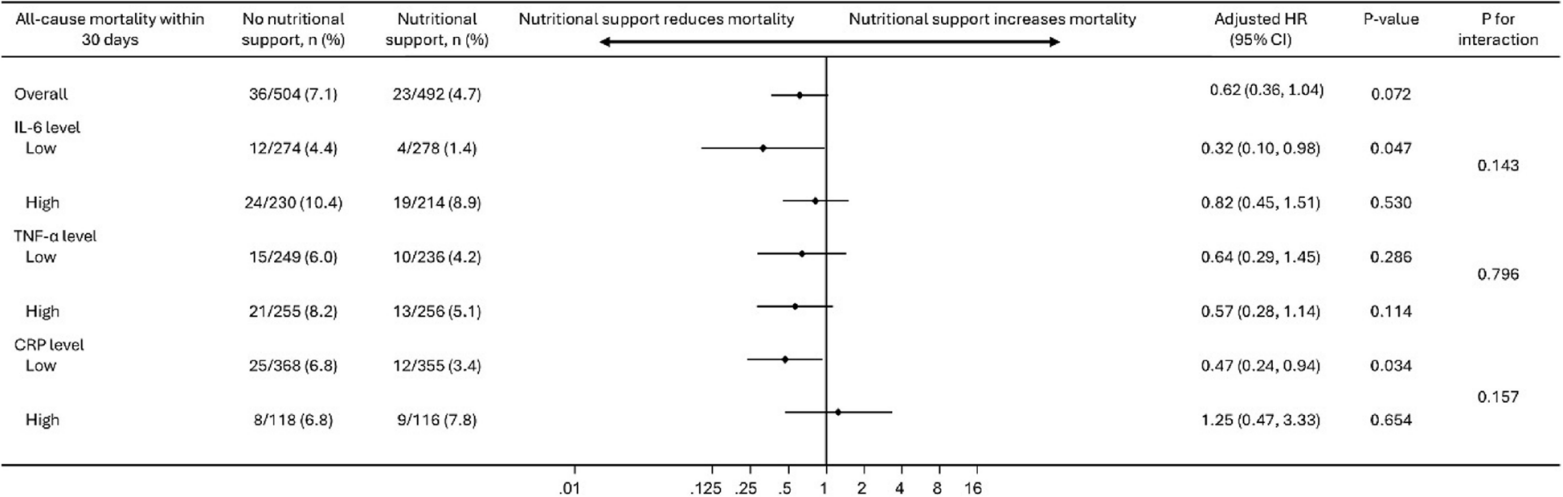
Forest Plot for therapy response comparing patients with high vs. low IL- 6, TNF-α and CRP levels with overall therapy response. HR, Hazard ratio, IL- 6, interleukin 6; TNF-α, tumor necrosis factor alpha; CRP, c-reactive protein

Next, we examined the effect of nutritional intervention on mortality by comparing patients in the intervention group with those in the control group, stratified by low versus high biomarker levels. Patients with elevated IL- 6 and CRP levels showed a less pronounced response to nutritional therapy in terms of mortality reduction compared to those with lower levels. Specifically, among patients with low IL- 6 levels, we found lower 30-day mortality in the intervention group (1.4%) compared with the control group (4.4%) with an adjusted HR of 0.32, 95% CI [0.10 to 0.98], *p* = 0.047. For patients with high IL- 6 levels, the benefit of nutritional therapy was not significant with an adjusted HR of 0.82 (95%CI 0.45 to 1.51, *p* = 0.530). Interaction analysis showed a trend, but not a significant difference (*p* = 0.143) (Fig. 2). In an exploratory analysis, we also stratified patients according to IL- 6 quartiles. The results showed that mainly patients with very high IL- 6 or very high CRP concentrations did not benefit from nutritional therapy, while patients in the lower three quartiles did (Supplemental Fig. 2). The evaluation of nutritional therapy in patients with very low IL6 or CRP is only possible to a limited extent due to the low event rate.

Results were similar also for CRP with a significant risk reduction for 30-day mortality in the intervention group for patients with low CRP levels at admission (adjusted HR 0.47, 95% CI 0.24 to 0.94, *p* = 0.034), but not for patients with high CRP levels (adjusted HR 1.25, 95% CI 0.47 to 3.33, *p* = 0.654) (p for interaction of 0.157). For TNF-α levels, however, stratified analyses did not display any difference in effect according to high compared to low admission levels.

## Discussion

Patients experiencing high levels of inflammation and associated metabolic stress may not gain the same benefits from nutritional therapy as those with lower levels of inflammation. Our analysis of three well-established inflammatory biomarkers—CRP, IL- 6, and TNF-α—as prognostic indicators of outcomes and treatment response in patients at nutritional risk confirmed this thesis and revealed two key findings: First, IL- 6 was a strong prognostic marker for short- and long-term mortality and further adverse outcomes in medical patients at nutritional risk. This was not found for CRP and TNF-α. Second, patients with high levels of IL- 6 and CRP had a less pronounced response to the individualized nutritional intervention compared to patients with low marker levels. This trend was not evident when patients were stratified by TNF-α levels.

We identified some distinct predictors for admission levels of IL- 6 and CRP, despite their physiological connection in sequence. A known trigger of elevated CRP levels is infection [29], which is also evident from our data, since infection as a main diagnosis is a strong predictor of admission CRP concentration. Although IL- 6 release induces hepatic production of CRP and thus both metabolites are interconnected, the main diagnosis of infection was not a significant predictor of IL- 6 admission concentration, suggesting a distinct spectrum of both cytokines. Additionally, CRP levels were higher in patients who reported a low food intake prior to hospitalization, which was not the case for IL- 6. This may be an unexpected finding as inflammation generally exerts important effects on metabolic and neuroendocrine functions, reducing appetite and affecting hunger regulation [6]. However, in our cohort of polymorbid inpatients, who were all at nutritional risk, mainly a higher disease severity was associated with higher IL- 6 levels. Differences in marker kinetics may also explain these observations.

In terms of prognostic value, IL- 6 was the only marker significantly associated with outcomes among our population of medical patients at nutritional risk. This is consistent with previous studies across various patient populations that also reported IL- 6 as a stronger prognostic marker for mortality and other outcomes with superior prognostic potential compared to CRP [30–32]. Possible explanations include the direct inflammatory effects of IL- 6 on specific organ systems [33, 34] and less variability of IL- 6 in the inflammatory pathway allowing for a more accurate assessment of the patient’s inflammatory burden. However, in contrast to the lack of association between CRP and adverse outcomes observed in our cohort, there are several studies demonstrating associations for both IL- 6 and CRP [30, 35]. Interestingly and in contrast to previous investigations in cancer patients [36], we did not find that TNF-α levels were associated with outcome in our sample. Differences in the study population and lower disease acuity in our cohort in relation to the very short half-life of 14 min of TNF-α may explain these differences [37]. 

Importantly, stratifying patients based on admission levels of IL- 6 and CRP enabled us to identify those with a less pronounced effect of nutritional therapy on mortality, confirming previous reports [16]. This is an important finding, as patients are typically selected for nutritional therapy based primarily on their risk of malnutrition rather than on the anticipated benefit of treatment. To date, this approach of treating at-risk patients independent of inflammation has been proven effective for medical inpatients [13]. However, as mentioned in the latest ESPEN guidelines on polymorbid patients [38] there is increasing evidence that phenotyping patients through the use of specific biomarkers may further improve treatment effects. Herein, this analysis supports these guidelines and suggests that patients with high inflammation [4] and high disease severity may require a different approach to reduce mortality [20, 39]. Although the pathophysiological concepts underlying this observation are still incomplete, we hypothesize that the presence of inflammatory cytokines directly affect skeletal muscle through various mechanisms, such as inhibition of muscle protein synthesis, increasing muscle catabolism through the ubiquitin-proteasomal system and increased autophagy, and impairment of myogenesis [40]. Next to the muscle-specific effects, inflammation also causes a loss of appetite, reduces intestinal motility and increases peripheral insulin resistance [6, 19]. This condition makes the patient particularly vulnerable to overfeeding [7, 14] especially when treated with full nutritional therapy – a phenomenon more commonly associated with ICU patients [41]. Therefore, easy-to-use and rapidly available biomarkers such as CRP and IL- 6 may be helpful in guiding nutritional therapy and a slower and more cautious approach to achieving nutritional targets may be advised in patients with high levels of inflammation to reduce mortality through nutritional therapy [42, 43].

## Strengths and limitations

This dataset originates from a randomized controlled trial involving a well-characterized cohort of polymorbid patients at nutritional risk, with prospectively measured short- and long-term outcomes. The analysis includes almost 1,000 patients from six different study sites, which increases external validity. Furthermore, we had complete data for three inflammatory markers and different baseline parameters for analysis. Nevertheless, there are certain limitations regarding the secondary analysis approach, selection bias and residual confounding. Furthermore, the study may have been underpowered for certain analyses, particularly regarding differences in outcomes, which means that the results are to be interpreted in an exploratory manner. Furthermore, we did not adjust for multiple testing. Additionally, treatment response was only assessed based on the effects of nutritional therapy on mortality and not on other outcomes, which only represents a part of the effect. The results of this analysis should be considered as generating hypotheses rather than definitive.

## Conclusion

Our findings support the concern that a high inflammatory state is associated with reduced benefit from nutritional therapy. Apparently, CRP and IL- 6 effectively predict treatment response, but IL- 6 may additionally serve as a prognostic marker for mortality. Further research is needed to improve treatment strategies for patients with elevated inflammatory profiles.

## Data Availability

Data described in the manuscript, code book, and analytic code will be made available to others with the publication of this manuscript, as already outlined in the primary EFFORT publication, on receipt of a letter of intention detailing the study hypothesis and statistical analysis plan. A signed data access agreement is required from all applicants. Please send requests to the principal investigator of this trial.

## SupplementaryInformation

Supplementary Material 1

## Abbreviations

IL- 6: Interleukin 6
TNFα: Tumor necrosis factor alpha
CRP: C-reactive protein
DRM: Disease-related malnutrition
EFFORT: Effect of early nutritional support on frailty, functional outcomes, and recovery of malnourished medical inpatients trial
CI: Confidence interval
OR: Odds ratio
HR: Hazard ratio
Coef.: Coefficient
SD: Standard deviation
p: p-value
N: Number of patients
ROC: Receiver operating characteristic
AUC: Area under the curve
RCT: Randomized controlled trial
NRS: Nutritional Risk Screening 2002
BMI: Body mass index
LOS: Length of hospital stay
ICU: Intensive care unit
COPD: Chronic obstructive pulmonary disease
