# Supplementary material for: Comparison of the inflammatory biomarkers IL- 6, TNF-α, and CRP to predict the effect of nutritional therapy on mortality in medical patients at risk of malnutrition: A secondary analysis of the randomized clinical trial EFFORT
Source: J Inflamm (Lond). 2025 Apr 24;22:16. doi: 10.1186/s12950-025-00442-0 (PMC12023447; doi:10.1186/s12950-025-00442-0)
Supplement: Supplementary file 1 — Supplementary Material 1 [file 12950_2025_442_MOESM1_ESM.docx]

**Comparison of the inflammatory biomarkers IL-6, TNF-α, and CRP to predict the effect of nutritional therapy on mortality in medical patients at risk of malnutrition**

***A secondary analysis of the randomized clinical trial EFFORT***

Carla Wunderle^1*^, Elisabeth Martin^1,2*^, Alma Wittig^1,2*^, Pascal Tribolet^1,3,4^, Thomas A. Lutz^5^, Christina Köster-Hegmann^5^, Zeno Stanga ^6^, Beat Mueller^1,2^, and Philipp Schuetz^1,2^

^1^Medical University Department, Division of General Internal and Emergency Medicine, Division of Endocrinology, Diabetes and Metabolism, Kantonsspital Aarau, 5001 Aarau, Switzerland

^2^Medical Faculty of the University of Basel, 4056 Basel, Switzerland

^3^Department of Health Professions, Bern University of Applied Sciences, 3008 Bern, Switzerland

^4^Department of Nutritional Sciences, Faculty of Life Sciences, University of Vienna, Josef-Holaubek-Platz 2, 1090 Vienna, Austria

^5^University of Zurich - Vetsuisse Faculty, Institute of Veterinary Physiology, 8057 Zurich, Switzerland

^6^Division of Diabetes, Endocrinology, Nutritional Medicine, and Metabolism, Bern University Hospital and University of Bern, 3010 Bern, Switzerland

Correspondence: [schuetzph@gmail.com](mailto:schuetzph@gmail.com)

*Equally contributing first authors

**Supplemental Table 1. Baseline characteristics, stratified by low and high TNF-α levels at admission**

|  |  | **Low TNF-α** | **High TNF-α** | **p-value** |
| --- | --- | --- | --- | --- |
| **Sociodemographic info** | |  |  |  |
|  | Patient number, n (%) | 485 (48.7) | 511 (51.3) |  |
|  | Male sex, n (%) | 275 (56.7) | 260 (50.9) | 0.066 |
|  | Age, mean (SD) | 73.3 (13.4) | 72.2 (15.3) | 0.23 |
| **Nutritional assessment, mean (SD)** | |  |  |  |
|  | Body mass index (kg/m²) | 24.7 (5.1) | 24.7 (5.4) | 0.86 |
|  | Mean body weight (kg) | 71.4 (16.5) | 70.3 (17.4) | 0.36 |
| **NRS total score, n (%)** | |  |  | 0.98 |
|  | 3 points | 163 (33.6) | 171 (33.5) |  |
|  | 4 points | 189 (39.0) | 194 (38.0) |  |
|  | 5 points | 113 (23.3) | 125 (24.5) |  |
|  | 6 points | 20 (4.1) | 21 (4.1) |  |
| **Intervention group** | | 236 (48.7) | 256 (50.1) | 0.65 |
| **Main diagnosis, n (%)** | |  |  |  |
|  | Infection | 141 (29.1) | 169 (33.1) | 0.17 |
|  | Cancer | 69 (14.2) | 73 (14.3) | 0.98 |
|  | Cardiovascular disease | 53 (10.9) | 56 (11.0) | 0.99 |
|  | Frailty | 55 (11.3) | 51 (10.0) | 0.49 |
|  | Lung disease | 34 (7.0) | 26 (5.1) | 0.20 |
|  | Gastrointestinal disease | 37 (7.6) | 54 (10.6) | 0.11 |
|  | Neurological disease | 37 (7.6) | 24 (4.7) | 0.054 |
|  | Renal disease | 6 (1.2) | 14 (2.7) | 0.091 |
|  | Metabolic disease | 13 (2.7) | 17 (3.3) | 0.55 |
|  | Other | 16 (3.3) | 8 (1.6) | 0.075 |
| **Comorbidity, n (%)** | |  |  |  |
|  | Hypertension | 243 (50.1) | 265 (51.9) | 0.58 |
|  | Malignant disease | 135 (27.8) | 148 (29.0) | 0.69 |
|  | Chronic kidney disease | 127 (26.2) | 158 (30.9) | 0.098 |
|  | Coronary heart disease | 132 (27.2) | 166 (32.5) | 0.07 |
|  | Diabetes | 111 (22.9) | 96 (18.8) | 0.11 |
|  | Congestive heart failure | 70 (14.4) | 98 (19.2) | 0.046 |
|  | COPD | 86 (17.7) | 74 (14.5) | 0.16 |
|  | Peripheral arterial disease | 41 (8.5) | 36 (7.0) | 0.41 |
|  | Stroke | 39 (8.0) | 35 (6.8) | 0.47 |
|  | Dementia | 16 (3.3) | 19 (3.7) | 0.72 |

We compared frequencies using Person`s chi-squared test and continuous, normally distributed variables using a two-sample t-test. TNF-α, tumor necrosis factor alpha; NRS, Nutritional Risk Screening 2002; COPD, chronic obstructive pulmonary disease

**Supplemental Table 2. Baseline characteristics, stratified by low and high CRP levels at admission**

|  |  | **Low CRP** | **High CRP** | **p-value** |
| --- | --- | --- | --- | --- |
| **Sociodemographic info** | |  |  |  |
|  | Patient number, n (%) | 723 | 234 |  |
|  | Male sex, n (%) | 383 (53.0) | 131 (56.0) | 0.42 |
|  | Age, mean (SD) | 72.3 (14.8) | 73.4 (13.2) | 0.33 |
| **Nutritional assessment, mean (SD)** | |  |  |  |
|  | Body mass index (kg/m²) | 24.6 (5.3) | 25.1 (5.1) | 0.23 |
|  | Mean body weight (kg) | 70.4 (17.5) | 72.4 (15.5) | 0.13 |
| **NRS total score, n (%)** | |  |  |  |
|  | 3 points | 264 (36.5) | 58 (24.8) | <0.001 |
|  | 4 points | 268 (37.1) | 101 (43.2) |  |
|  | 5 points | 169 (23.4) | 57 (24.4) |  |
|  | 6 points | 22 (3.0) | 18 (7.7) |  |
| **Intervention group** | | 355 (49.1) | 116 (49.6) | 0.90 |
| **Main diagnosis, n (%)** | |  |  |  |
|  | Infection | 145 (20.1) | 154 (65.8) | <0.001 |
|  | Cancer | 103 (14.2) | 30 (12.8) | 0.58 |
|  | Cardiovascular disease | 98 (13.6) | 7 (3.0) | <0.001 |
|  | Frailty | 92 (12.7) | 11 (4.7) | <0.001 |
|  | Lung disease | 48 (6.6) | 9 (3.8) | 0.12 |
|  | Gastrointestinal disease | 81 (11.2) | 8 (3.4) | <0.001 |
|  | Neurological disease | 56 (7.7) | 3 (1.3) | <0.001 |
|  | Renal disease | 16 (2.2) | 3 (1.3) | 0.37 |
|  | Metabolic disease | 29 (4.0) | 0 (0.0) | 0.002 |
|  | Other | 16 (2.2) | 5 (2.1) | 0.94 |
| **Comorbidity, n (%)** | |  |  |  |
|  | Hypertension | 365 (50.5) | 116 (49.6) | 0.81 |
|  | Malignant disease | 200 (27.7) | 72 (30.8) | 0.36 |
|  | Chronic kidney disease | 203 (28.1) | 68 (29.1) | 0.77 |
|  | Coronary heart disease | 218 (30.2) | 64 (27.4) | 0.41 |
|  | Diabetes | 142 (19.6) | 52 (22.2) | 0.39 |
|  | Congestive heart failure | 128 (17.7) | 34 (14.5) | 0.26 |
|  | COPD | 119 (16.5) | 37 (15.8) | 0.82 |
|  | Peripheral arterial disease | 59 (8.2) | 14 (6.0) | 0.28 |
|  | Stroke | 53 (7.3) | 18 (7.7) | 0.85 |
|  | Dementia | 25 (3.5) | 10 (4.3) | 0.56 |

We compared frequencies using Person`s chi-squared test and continuous, normally distributed variables using a two-sample t-test. CRP, C-reactive protein; NRS, Nutritional Risk Screening 2002; COPD, chronic obstructive pulmonary disease

**Supplemental Table 3. Association of patient characteristics and nutritional parameters with IL-6, TNF-α and CRP**

|  |  | **IL-6** | | **TNF-α** | | **CRP** | | |
| --- | --- | --- | --- | --- | --- | --- | --- | --- |
|  |  | **Coeff (95% CI)** | **p-value** | **Coeff (95% CI)** | **p-value** | **Coeff (95% CI)** | | **p-value** |
| ***Sociodemographic predictors*** | | | |  |  |  | | |
| Male sex | | 3.01 (-0.38 to 6.39) | 0.082 | -1.10 (-1.92 to -0.28) | 0.009 | 7.92 (-1.45 to 17.28) | 0.097 | |
| Age years, mean (SD) | | 0.01 (-0.11 to 0.13) | 0.884 | -0.02 (-0.05 to 0.01) | 0.157 | 0.33 (0.00 to 0.66) | 0.050 | |
| **Nutritional parameters** | |  |  |  |  |  |  | |
| Body mass index (kg/m²) | | -0.08 (-0.41 to 0.25) | 0.634 | 0.02 (-0.06 to 0.10) | 0.555 | 0.16 (-0.76 to 1.07) | 0.738 | |
| Weight loss | |  |  |  |  |  |  | |
| <5% in 3 months | | reference |  | reference |  | reference |  | |
| >5% in 3 months | | -0.94 (-5.16 to 3.28) | 0.662 | 0.44 (-0.58 to 1.47) | 0.397 | 4.13 (-7.55 to 15.81) | 0.488 | |
| >5% in 2 months | | -2.59 (-8.25 to 3.07) | 0.370 | -0.26 (-1.63 to 1.12) | 0.714 | -16.83 (-32.60 to -1.06) | 0.036 | |
| >5% in 1 months | | -1.79 (-6.52 to 2.95) | 0.460 | 0.79 (-0.36 to 1.94) | 0.178 | -9.88 (-22.85 to 3.09) | 0.135 | |
| Loss of appetite | | 1.71 (-6.89 to 10.31) | 0.697 | -0.19 (-2.28 to 1.90) | 0.857 | -1.34 (-25.36 to 22.67) | 0.913 | |
| Reduced dietary intake | |  |  |  |  |  |  | |
| 1 = Reference > 75% | | reference |  | reference |  | reference |  | |
| 2 = 50-75% | | 3.96 (-5.06 to 12.98) | 0.389 | 0.44 (-1.76 to 2.63) | 0.696 | 26.04 (0.94 to 51.14) | 0.042 | |
| 3 = 25-50% | | 2.70 (-6.60 to 12.00) | 0.569 | -0.14 (-2.39 to 2.12) | 0.906 | 35.59 (9.60 to 61.57) | 0.007 | |
| 4 = < 25% | | 4.82 (-5.16 to 14.81) | 0.343 | 1.18 (-1.24 to 3.60) | 0.340 | 41.93 (14.19 to 69.66) | 0.003 | |
| **Disease severity** | |  |  |  |  |  |  | |
| 1 | | reference |  | reference |  | reference |  | |
| 2 | | 5.99 (2.00 to 9.97) | 0.003 | -0.07 (-1.03 to 0.90) | 0.894 | 35.97 (24.92 to 47.02) | < 0.001 | |
| 3 | | 37.26 (19.54 to 54.98) | < 0.001 | 0.71 (-3.38 to 4.80) | 0.732 | 31.64 (-18.93 to 82.20) | 0.220 | |
| ***Clinical predictors*** | |  |  |  |  |  |  | |
| **Main diagnosis** | |  |  |  |  |  |  | |
| Infectious disease | | 6.22 (-2.43 to 14.88) | 0.159 | -0.75 (-2.83 to 1.33) | 0.479 | 73.42 (52.07 to 94.78) | < 0.001 | |
| Cancer | | -4.73 (-14.46 to 5.00) | 0.340 | -2.62 (-4.96 to -0.28) | 0.028 | 13.94 (-10.18 to 38.06) | 0.257 | |
| Cardiovascular disease | | 1.34 (-8.51 to 11.18) | 0.790 | -2.03 (-4.39 to 0.34) | 0.093 | -5.98 (-30.38 to 18.43) | 0.631 | |
| Frailty | | -4.73 (-14.31 to 4.85) | 0.333 | -1.98 (-4.28 to 0.33) | 0.093 | -1.59 (-25.26 to 22.08) | 0.895 | |
| Lung disease | | -1.13 (-12.04 to 9.77) | 0.838 | -1.66 (-4.28 to 0.95) | 0.213 | 10.23 (-16.87 to 37.33) | 0.459 | |
| Gastrointestinal disease | | 0.46 (-9.33 to 10.25) | 0.926 | 0.59 (-1.76 to 2.95) | 0.621 | -3.52 (-27.67 to 20.62) | 0.775 | |
| Neurological disorders | | -5.51 (-16.24 to 5.21) | 0.313 | -1.67 (-4.25 to 0.91) | 0.204 | -21.8 (-48.39 to 4.79) | 0.108 | |
| Renal disease | | -3.26 (-17.48 to 10.96) | 0.653 | -1.18 (-4.60 to 2.24) | 0.498 | 1.43 (-35.00 to 37.85) | 0.939 | |
| Metabolic disease | | -5.88 (-18.39 to 6.63) | 0.357 | -1.45 (-4.46 to 1.56) | 0.344 | -23.79 (-54.95 to 7.37) | 0.134 | |
| Other | | -3.89 (-17.29 to 9.52) | 0.569 | -1.84 (-5.07 to 1.38) | 0.263 | 9.62 (-25.52 to 44.75) | 0.591 | |
| **Comorbidities** | |  |  |  |  |  |  | |
| Hypertension | | -0.90 (-4.58 to 2.79) | 0.633 | 0.16 (-0.72 to 1.05) | 0.72 | -1.97 (-11.28 to 7.35) | 0.678 | |
| Malignant disease | | 7.29 (3.03 to 11.55) | 0.001 | 1.89 (0.87 to 2.91) | < 0.001 | 9.10 (-1.72 to 19.92) | 0.099 | |
| Renal insufficiency | | 2.19 (-1.84 to 6.22) | 0.287 | -0.19 (-1.16 to 0.78) | 0.701 | 7.06 (-3.22 to 17.33) | 0.178 | |
| Coronary heart disease | | 3.03 (-1.14 to 7.20) | 0.154 | 1.68 (0.68 to 2.68) | 0.001 | -2.00 (-12.69 to 8.70) | 0.714 | |
| Diabetes mellitus | | 0.38 (-3.91 to 4.66) | 0.863 | -0.71 (-1.73 to 0.32) | 0.175 | 4.30 (-6.60 to 15.20) | 0.439 | |
| Congestive heart failure | | 0.06 (-5.08 to 5.19) | 0.983 | 0.81 (-0.43 to 2.04) | 0.198 | -2.47 (-15.51 to 10.58) | 0.711 | |
| COPD | | -3.20 (-8.16 to 1.76) | 0.205 | -0.82 (-2.01 to 0.37) | 0.178 | 0.77 (-11.77 to 13.31) | 0.904 | |
| Peripheral artery disease | | 1.70 (-4.78 to 8.19) | 0.606 | -0.35 (-1.90 to 1.21) | 0.661 | -7.95 (-24.49 to 8.59) | 0.346 | |
| Stroke | | -0.20 (-6.87 to 6.46) | 0.952 | -0.52 (-2.13 to 1.08) | 0.523 | -5.87 (-22.85 to 11.12) | 0.498 | |
| Dementia | | -0.85 (-10.03 to 8.32) | 0.856 | 0.40 (-1.81 to 2.61) | 0.723 | -2.74 (-25.52 to 20.03) | 0.813 | |
|  | |  |  |  |  |  | | |

Coefficients were calculated with a linear regression. We used two multivariate models: sex, age, body mass index, weight loss, loss of appetite, reduced dietary intake, disease severity, and sex, age, main diagnoses, comorbidities. The nutritional parameters are categorical variables. IL-6, interleukin 6; TNF-α, tumor necrosis factor alpha; CRP, C-reactive protein; CI, confidence interval; Coeff, coefficient; COPD, chronic obstructive pulmonary disease.

**Supplemental Table 4. Prognostic value of IL-6, TNF-α and CRP on secondary outcomes**

|  |  | **IL-6** | | **TNF-α** | | **CRP** | |
| --- | --- | --- | --- | --- | --- | --- | --- |
| **Clinical outcomes** | | **adj. OR (95% CI)** | **p-value** | **adj. OR (95% CI)** | **p-value** | **adj. OR (95% CI)** | **p-value** |
| Major complications | | 1.63 (1.01 to 2.64) | 0.046 | 1.13 (0.71 to 1.80) | 0.611 | 1.32 (0.73 to 2.37) | 0.362 |
| Adverse events | | 1.34 (0.98 to 1.83) | 0.066 | 1.03 (0.76 to 1.41) | 0.829 | 1.08 (0.72 to 1.62) | 0.715 |
| ICU | | 1.70 (0.75 to 3.82) | 0.201 | 1.51 (0.67 to 3.39) | 0.315 | 1.00 (0.35 to 2.85) | 0.994 |
| Rehospitalization | | 0.63 (0.39 to 1.02) | 0.063 | 0.85 (0.54 to 1.33) | 0.466 | 0.71 (0.37 to 1.37) | 0.308 |
| Bartel decline of 10 percent | | 2.16 (1.39 to 3.34) | 0.001 | 1.00 (0.66 to 1.53) | 0.983 | 1.26 (0.73 to 2.17) | 0.407 |
| Nosocomial infection | | 1.30 (0.71 to 2.37) | 0.400 | 1.30 (0.71 to 2.36) | 0.392 | 0.97 (0.46 to 2.05) | 0.929 |
| Respiratory insufficiency | | 1.18 (0.44 to 3.18) | 0.748 | 1.71 (0.62 to 4.70) | 0.299 | 1.74 (0.51 to 5.92) | 0.377 |
| **Further secondary outcomes** | | **Coeff (95% CI)** | **p-value** | **Coeff (95% CI)** | **p-value** | **Coeff (95% CI)** | **p-value** |
| Length of stay | | 1.81 (0.95 to 2.66) | <0.001 | 0.39 (-0.45 to 1.23) | 0.365 | 1.53 (0.44 to 2.63) | 0.006 |
| Quality of life | |  |  |  |  |  |  |
| Visual analogue scale | | -0.77 (-3.93 to 2.38) | 0.631 | 0.93 (-2.08 to 3.95) | 0.543 | 0.38 (-3.61 to 4.37) | 0.853 |
| EQ-5D | | -1.40 (-4.01 to 1.21) | 0.294 | -1.76 (-4.30 to 0.78) | 0.174 | -3.20 (-6.54 to 0.13) | 0.060 |

HR >1, as well as OR >1 indicate a positive association of high cytokine plasma levels with the outcomes. We adjusted for randomization group, sex, NRS, age, the four most frequent main diagnoses (cancer, infection, cardiovascular disease, frailty), the two most frequent comorbidities (hypertension, renal insufficiency). IL-6, interleukin 6; TNF-α, tumor necrosis factor alpha; CRP, C-reactive protein; CI, confidence interval; HR, hazard ratio; OR, odds ratio; Coeff, coefficient; ICU, intensive care unit; NRS, nutritional risk screening 2002

**Supplemental Figure 1. Study flow chart**

**
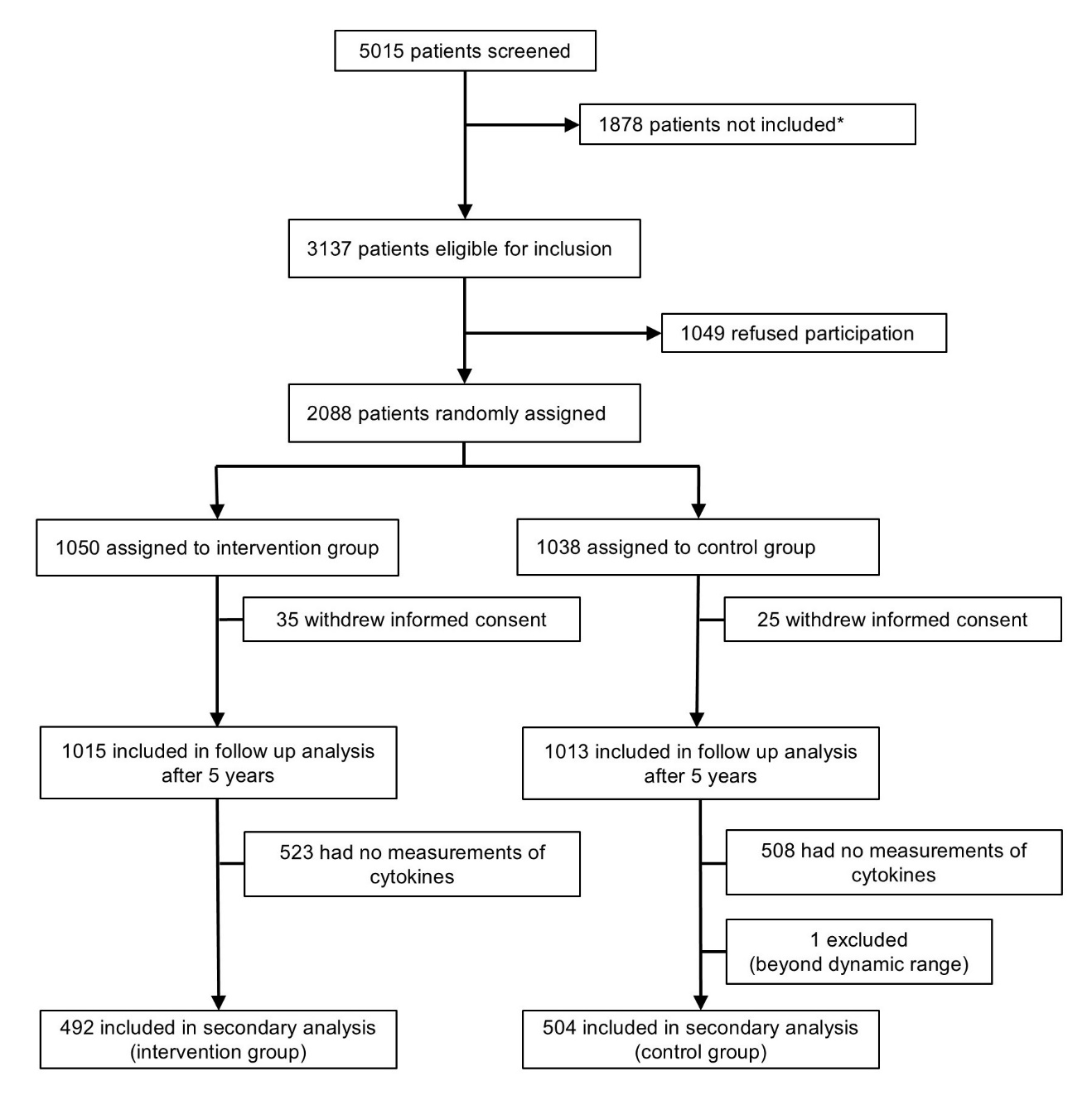
**

*Reasons for exclusion of patients: 145 surgical patients; 268 unable to ingest oral nutrition; 158 with a terminal condition; 719 patients already receiving nutritional therapy on admission; 31 hospitalized because of anorexia nervosa; 161 with acute pancreatitis; 81 with acute liver failure; 6 with cystic fibrosis; 11 after stem cell transplantation; 27 with malnutrition after gastric bypass operations; 43 with contraindication against nutritional therapy; 228 previously included in the trial

**Supplemental Figure 2. Forest Plot for therapy response comparing quartiles of IL-6 and CRP levels with overall therapy response**

**
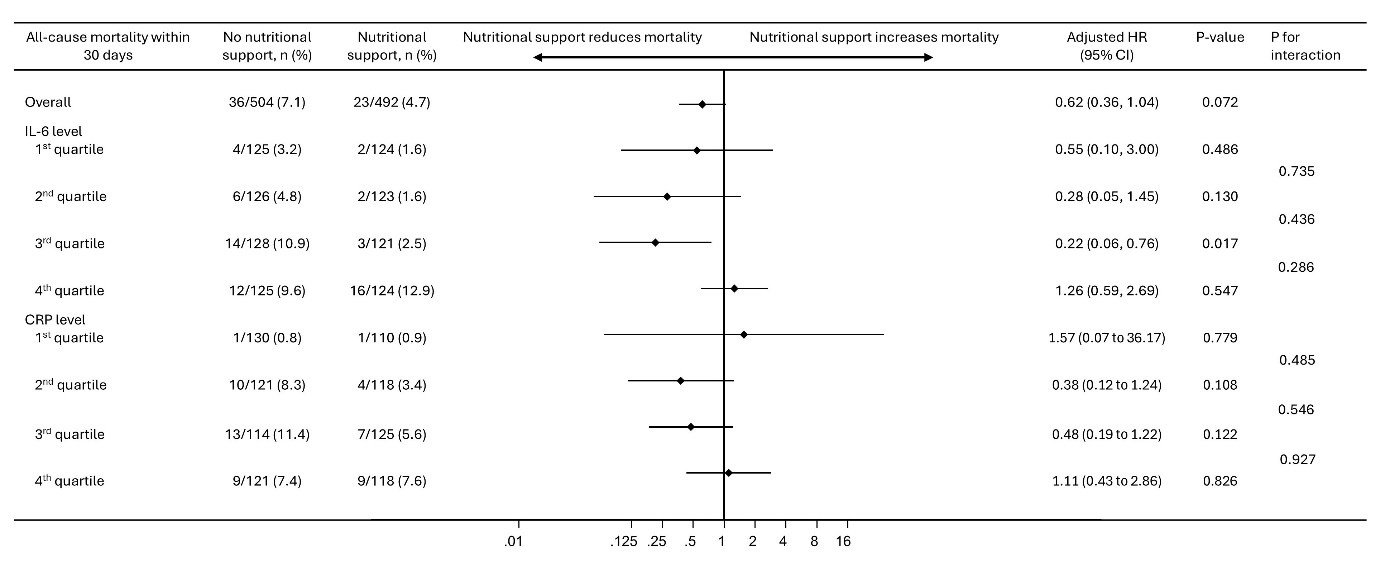
**

Quartiles of IL-6 : <5.32 pg/mL, 5.32-9.79 pg/mL, 9.79-19.79 pg/mL, >19.79 pg/mL. Quartiles of CRP : <6.9 mg/L, 6.9-30.8 mg/L 30.8-97 mg/L, >97 mg/L. HR, Hazard ratio, IL-6, interleukin 6; TNF-α, tumor necrosis factor alpha; CRP, c-reactive protein.
